# Symptoms, Imaging Features, Treatment Decisions, and Outcomes of Patients with Top of the Basilar Artery Syndrome: Experiences from a Comprehensive Stroke Center

**DOI:** 10.1007/s12028-025-02219-y

**Published:** 2025-02-07

**Authors:** Franziska Lieschke, Maximilian Rauch, Bastian Roller, Jan Hendrik Schaefer, Martin A. Schaller-Paule

**Affiliations:** 1 Department of Neurology, University Hospital Frankfurt, Goethe-University, Frankfurt Am Main, Germany; 2 Institute of Neuroradiology, University Hospital Frankfurt, Goethe-University, Frankfurt Am Main, Germany; 3 Practice for Neurology and Psychiatry Eltville, Eltville, Germany

**Keywords:** Top of the basilar artery syndrome, Basilar occlusion, Acute ischemic stroke, Posterior circulation, Mechanical thrombectomy

## Abstract

**Background:**

From visual, ocular, and pupillomotor abnormalities to qualitative and more importantly rapid quantitative disturbances of consciousness, top of the basilar artery syndrome (TOBS) represents a diagnostic challenge in neurocritical care. In this monocentric retrospective cross-sectional study, we will describe this particular patient group in detail and highlight its variability and the associated implications.

**Methods:**

Consecutive patients with radiologically confirmed TOBS presenting to our comprehensive stroke center were analyzed from 2010 to 2022. Baseline parameters at admission, including clinical symptoms, National Institutes of Health Stroke Scale (NIHSS) score, Glasgow Coma Scale (GCS) score, and imaging parameters (mode and success of recanalization measured by the Thrombolysis in Cerebral Infarction [TICI] score, extent of infarct, and infarct localization), were assessed. Functional dependence at discharge was analyzed with the modified Rankin scale (mRS) and Barthel Index.

**Results:**

We assessed 96 eligible patients with a mean age of 70 (SD ± 14) years, 41.67% of whom were female. The median NIHSS score at admission was 19 (interquartile range [IQR] 8–35), and the median GCS score was 7 (IQR 3–15). Dysphagia was identified in 51.72% of patients, with a significant number discharged with nasogastric tubes. Most patients received both intravenous thrombolysis (IVT) and mechanical thrombectomy (MT) (47%), whereas 32% received MT only, and 10% received no acute recanalizing therapy. Patients receiving both IVT and MT had higher frequencies of successful vessel revascularization (higher TICI scores) and better clinical outcomes compared to those receiving only MT (median mRS score 4 [IQR 2–5] vs. 5 [IQR 2–6], *p* = 0.046). Multivariable regression analysis confirmed that successful recanalization (TICI) and GCS score at admission were key predictors of functional outcomes.

**Conclusions:**

A large proportion of patients presenting with TOBS were severely affected by a significant reduction in vigilance, a condition that persists in the absence of recanalization and is then associated with a relevant dependency.

**Supplementary Information:**

The online version contains supplementary material available at 10.1007/s12028-025-02219-y.

## Introduction

Posterior circulation strokes account for up to 25% [1, 2] of all ischemic strokes and are linked to high rates of recurrence, disability, and mortality [3, 4]. Clinical diagnosis is often challenging because of unspecific symptoms such as dizziness, sometimes headache, nausea and vomiting, or mental status alterations, which reduce public awareness [5]. Computed tomography (CT) has limitations in detecting posterior fossa strokes, making magnetic resonance imaging (MRI) the preferred modality [6]. However, 20–30% of posterior circulation strokes may appear negative on diffusion-weighted imaging within the first 12 h [7–9]. Consequently, a high percentage is misdiagnosed [5], onset to intravenous thrombolysis (IVT)/onset to groin puncture times are longer, and overall IVT rates are lower in posterior circulation strokes, leading to higher mortality and poor functional outcome [10].

Top of the basilar artery syndrome (TOBS) is particularly difficult to diagnose, arising from occlusion of the distal third of the basilar artery [11] or, rarely, compression from unruptured aneurysms at the basilar apex if the aneurysm compresses the posterior thalamoperforating arteries, as first described by Prof. Castaigne in 1981 [12]. Symptoms include sudden loss of consciousness; visual, oculomotor and pupillomotor, and behavioral abnormalities (mnestic dysfunction, agitated behavior, vivid hallucinations, and sometimes dreamlike behavior); and ataxia, often without major motor or sensory deficits [11, 13]. Symptoms vary depending on the length and position of the clot, the extent of ischemia, and collateral flow. Particularly the presence of a posterior communicating artery is favorable [14]. Thus, the diagnosis is often made based on the neuroradiological findings and goes along with infarction of the rostral brainstem, medial temporal and/or occipital lobes, thalamus, and cerebellum. IVT is often effective, whereas the use of thrombectomy is discussed controversially [15]. Recent evidence supports mechanical thrombectomy (MT) as a safe option for posterior circulation large vessel occlusion strokes [16–20] (Table 1), though its superiority over IVT, especially in mild cases, is unclear. This retrospective, monocentric cross-sectional study aimed to identify clinical and imaging factors influencing treatment decisions and clinical outcome in patients with TOBS who presented to our comprehensive stroke center. We systematically analyzed clinical presentation, imaging findings, and treatments, hypothesizing that mesencephalic involvement predicts poorer outcomes.

**Table 1:** Large clinical trials investigating the role of MT in posterior circulation large vessel occlusion stroke

Study name and trial enrollment period	Study design	Reported main finding
BEST [15], April 2015 to September 2017	RCT comparing MT + best medical care (IVT if applicable) vs. best medical care alone < 8 h of symptom onset. Note: terminated due to loss of equipoise (high crossover rate, small sample size)	No difference in favorable outcomes
BASILAR [16], January 2014 to May 2019	Nonrandomized cohort study comparing MT + best medical care (IVT if applicable) vs. best medical care alone < 24 h of symptom onset	Better functional outcomes and reduced mortality in MT group
BASICS [17], October 2011 to December 2019	RCT comparing MT + best medical care (IVT if applicable) vs. best medical care alone < 6 h of symptom onset	No difference in favorable outcome but trend toward benefit of MT for severely affected patients (NIHSS > 10)
BAOCHE [18], August 2016 to June 2021	RCT comparing MT + best medical care (IVT if applicable) vs. best medical care alone within 6 to 24 h after symptom onset	Better functional outcome at 90 days but more procedural complications and more cerebral hemorrhages in MT group
ATTENTION [19], February 2021 to March 2021	RCT within 12 h after symptom onset	Better functional outcome, lower mortality for patients receiving MT + BMT compared to BMT up to 24 h from symptom onset

ATTENTION, Endovascular Treatment for Acute Basilar Artery Occlusion; BAOCHE, Basilar Artery Occlusion Chinese Endovascular Trial; BASICS, Basilar Artery International Cooperation Study; BASILAR, Endovascular Treatment for Acute Basilar Artery Occlusion Study; BEST, Basilar Artery Occlusion Endovascular Intervention vs. Standard medical Treatment; BMT, best medical treatment; IVT, intravenous thrombolysis; MT, mechanical thrombectomy; NIHSS, National Institutes of Health Stroke Scale; RCT, randomized controlled trial

## Methods

In this retrospective single-center cross-sectional study, a systematic radiological database search including all brain scans (CT and MRI) conducted between 2010 and 2022 was performed based on search terms related to distal basilar artery occlusion and TOBS (translated from German: distal basilar artery thrombosis, distal basilar artery syndrome, thrombus of the distal basilar artery, thrombus in the distal basilar artery, distal basilar thrombus, distal basilar thrombosis). In a next step, we checked for sufficient image quality and completeness of the data records for evaluation. In a third step (after removal of duplicates), we selected patients with TOBS based on the predefined thrombus location (occlusion of the distal third of the basilar artery).

The following clinical baseline parameters and demographic data were captured: age, sex, information on previous diseases and vascular risk factors, modified Rankin scale (mRS) score before admission, National Institutes of Health Stroke Scale (NIHSS) score at admission and at discharge, stroke etiology according to Trial of Org 10,172 in Acute Stroke Treatment (TOAST) classification, treatment with IVT and/or MT, duration between symptom onset and arrival in hospital, presenting symptoms (such as dysarthria, dysphagia, hemiparesis, hemisensory loss, ataxia, vertigo, bulbar, hemianopia, pupillomotor, oculomotor, and Babinski sign), and vigilance at admission (according to the Glasgow Coma Scale [GCS]). Dysphagia was confirmed via clinical swallowing assessment with or without flexible endoscopic evaluation of swallowing (FEES) by an experienced speech and language therapist according to our standardized protocol. Every stroke patient who is not intubated and ventilated is seen once by a speech therapist within the first 24 h after admission. Initially, the patient’s level of alertness is assessed, and if the patient is sufficiently alert, a basic clinical swallowing examination is performed. If this examination yields critical findings (i.e., neurogenic dysphagia is suspected), FEES is subsequently conducted to confirm the diagnosis and test suitable nutritional regimens. Detailed documentation was kept regarding why FEES examinations were not performed for certain patients.

All radiological parameters were obtained by two experienced neuroradiologists and captured as follows: posterior circulation Alberta Stroke Program Early CT Score (pcASPECTS) on initial imaging, thrombus length in millimeters, involvement and localization of additional vessel occlusions (right/left P1 segment of the posterior cerebral artery, right/left superior cerebellar artery [SUCA]), recanalization success (according to the Thrombolysis in Cerebral Infarction [TICI] score), and infarct localization (thalamic, occipital, cerebellar, mesencephalic, and pontine). If discrepancies occurred, the cases were revised together and both radiologists agreed directly on a result. Outcome was focused on functional independence (mRS and Barthel Index [BI]) at discharge. The study was approved by the local ethics committee (No. 20–616).

Data were analyzed using GraphPad Prism 10 (Version 10.2.2) and GNU R 4.4.1. Ordinal and nonnormal data are presented as medians with interquartile ranges (IQRs), and normally distributed data are presented as mean ± SEM or SD. Nominal data are expressed as percentages. Categorical data were evaluated for differences by the χ^2^ test or Fisher’s exact test, with odds ratios and 95% confidence intervals calculated from contingency tables. Metric data were assessed by Student’s *t*-test or the Mann–Whitney *U*-test depending in normality (Kolmogorov–Smirnov test). Comparisons between more than two groups were performed using the Kruskal–Wallis test and Dunn’s multiple comparison test. Univariate analysis used Spearman correlation (*ρ*) with false discovery rate (FDR) corrections via the Benjamini–Hochberg procedure (Web-based FDR calculator from DMCA 2016 Carbocation Corporation). To model functional outcome, a multivariable median regression was performed. The significance level for all tests was set at *p* < 0.05.

## Results

**Fig. 1:**
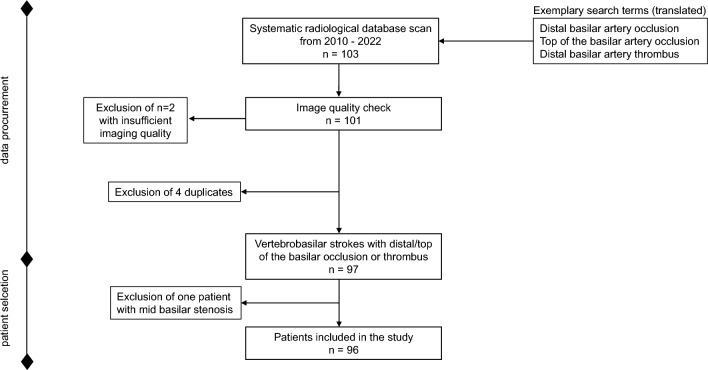
Study flowchart describing the data procurement and stepwise patient selection process intended for retrospective analysis. Further search terms were (translated from German): distal basilar artery thrombosis, distal basilar artery syndrome, thrombus of the distal basilar artery, thrombus in the distal basilar artery, distal basilar thrombus, and distal basilar thrombosis

Applying the prespecified search term, 103 patients were identified. Two cases were excluded for poor image quality. After subtracting four duplicates and one patient who did not meet the TOBS definition, 96 patients were finally included in the study (Fig. 1).

### Clinical Findings at Admission

The mean age was 70.32 years, and 41.67% of the patients were female. Seventy-two percent of the patients presented with a decreased level of consciousness, along with bulbar symptoms (dysphagia in 61%, dysarthria in 80%), hemiparesis (54%), pupillomotor deficits (46%), and oculomotor deficits (50%). The Babinski sign was positive on at least one side in 34% of the cases. The median NIHSS score at admission was 19, whereas the median prior mRS score was 1. In total, 33 patients (31%) presented with prior antiplatelet monotherapy (32 patients with aspirin, 1 with clopidogrel) and 5 patients (5%) presented with dual antiplatelet therapy, all of them due to recent cardiac interventions (coronary stents). Seventeen patients (18%) presented with prior oral anticoagulation, of whom 12 patients received the vitamin K antagonist phenprocoumon and 5 patients received direct oral anticoagulants. For more baseline characteristics, see Table 2.

**Table 2:** Patient baseline characteristics

Patient characteristics	*n*	%	Mean	SD	Median	IQR
Total	96					
Age, y			70.32	14.38		
*Sex*						
Male	56	58				
Female	40	42				
*Stroke risk factors*						
Arterial hypertension	79	82				
Diabetes mellitus	26	27				
Hypercholesterinemia	71	74				
Atrial fibrillation	51	53				
*Prior medication*						
Unknown	9	9				
No AP/no OAC	30	31				
AP	33	34				
OAC	17	18				
AP + OAC	2	2				
DAPT	5	5				
NIHSS at admission					19	8–35
*Prior mRS*						
Overall					0.5	0–1
mRS 0	48	50				
mRS 1	26	27				
mRS 2	12	13				
mRS 3	9	9				
mRS 4	1	1				
GCS at admission					7	3–15
*Vigilance*						
Awake	27	28				
Somnolent	17	18				
Stuporous	6	6				
Comatose	8	8				
Analgosedated	38	40				
*Symptoms*						
Dysarthria	77	80				
Dysphagia	59	61				
Hemiparesis	52	54				
Hemisensory loss	16	17				
Ataxia	38	40				
Vertigo	29	30				
Bulbar	10	10				
Hemianopia	14	15				
Pupillomotor	44	46				
Oculomotor	48	50				
Babinski sign	33	34				
*Imaging*						
CT	60	62.5				
MRI	36	37.5				
Onset-to-door time (minutes)	75		244.2	180.9	221	90–325
Treatment with IVT (minutes)	56	58				
Onset-to-needle time	14	25	178.7	81.0	194.5	100–240
Door-to-needle time	17	30	68.7	58.6	44	20–100
Treatment with MT	75	78				
*TOAST etiology*						
Atherothrombotic	24	25				
Cardioembolic	53	55				
Small vessel	0	0				
Other determined	7	7				
Undetermined	12	13				
*Secondary prevention*						
Unchanged	18	19				
Start OAC	23	24				
Change OAC	8	8				
Start AP	17	18				
Change AP/start DAPT	13	14				

AP, antiplatelet therapy; CT, computed tomography; DAPT, dual antiplatelet therapy; GCS, Glasgow Coma Scale; IVT, intravenous thrombolysis; MRI, magnetic resonance imaging; mRS, modified Rankin scale; MT, mechanical thrombectomy; NIHSS, National Institutes of Health Stroke Scale; OAC, oral anticoagulation; TOAST, Trial of Org 10,172 in Acute Stroke Treatment

### Radiological Findings at Admission

**Fig. 2:**
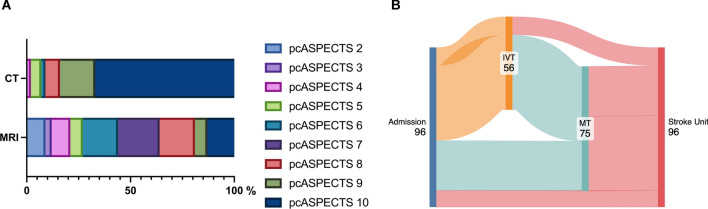
**a**, Frequency distribution of the pcASPECTS in the initial imaging (CT group *n* = 60 vs. MRI group *n* = 36; *p* < 0.0001). **b**, Sankey diagram of treatments. Made with SankeyMATIC.com. CT computed tomography, IVT intravenous thrombolysis, MRI magnetic resonance imaging, MT mechanical thrombectomy, pcASPECTS posterior circulation Alberta Stroke Program Early CT Score

The mean length of the thrombi was 9.6 mm (± 6.5 mm) and most frequently included an additional occlusion of the P1 segments with slightly more frequent involvement of the left side (Supplemental Table S1). Overall, 46 patients (49%) did not show any infarcts in the initial imaging. Infarction of the thalamus was found unilaterally in 13 (13.7%) and bilaterally in 7 cases (7.4%). In addition, 13 patients (13.7%) had unilateral occipital infarcts, and 2 (2.1%) had bioccipital infarcts. Overall, 22 patients (23.2%) showed infarcts of the mesencephalon, 17 (17.9%) showed infarcts of the pons, 19 (20%) had unilateral cerebellar infarct, and 11 (11.6%) had bilateral cerebellar infarcts. MRI provided superior infarct delineation compared to CT, with significantly lower pcASPECTS scores (median 7 [IQR 5–8] for MRI vs. 10 [IQR 9–10] for CT, *p* < 0.0001; Fig. 2a).

One patient was identified in whom a partial thrombosis of an unruptured giant basilar artery aneurysm caused TOBS (representing Castaigne syndrome). After a thorough workup, we assumed that a carryover of thrombotic material from the aneurysm into both P1 segments and both SUCAs caused an extended infarction (bioccipital, bimesencephalic, bicerebellar, left pontine, and left thalamic).

### Treatment Decisions

In our cohort, 11 patients (11%) received IVT only, 45 patients additionally received MT (IVT + MT 47%), 30 patients received MT without thrombolysis (32%), and 10 patients received no acute recanalizing therapy in the form of MT or thrombolysis (10%; Fig. 2b). Time windows from symptom onset to arrival at our comprehensive stroke center were not statistically significantly different between the treatment groups (Kruskal–Wallis test *p* = 0.5877; Supplemental Table S2). IVT initiation was often precluded by preexisting infarct demarcation (in 52.2% of the cases), prior anticoagulation, time window limitations, or bleeding risks (for details, see Supplemental Table S3). Anticoagulation or antiplatelet therapy was adjusted based on individual risk profiles, including atrial fibrillation detection and stent placement. Detailed information on the secondary prevention strategies can be found in the supplementary material.

### Reperfusion Status

**Fig. 3:**
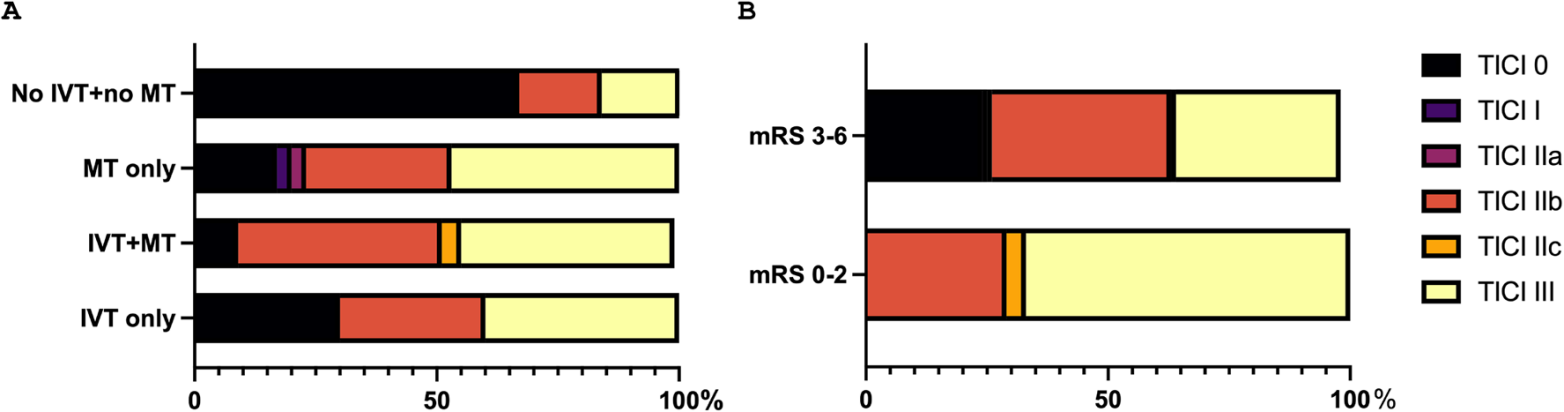
**a**, Frequency distribution of the reperfusion status (represented by mTICI score) in the individual treatment groups (IVT only group: 30% TICI 0, 30% TICI IIb, 40% TICI III; IVT + MT group: 9% TICI 0, 42% TICI IIb, 4% TICI IIc, 44% TICI III; MT only group: 17% TICI 0, 3% TICI I, 3% TICI IIa, 30% TICI IIb, 47% TICI III; no IVT + no MT group: 67% TICI 0, 17% TICIIIb, 17% TICI III; *p* < 0.0001). **b**, Frequency distribution of the reperfusion status in the dependent (mRS 3–6) vs. the independent (mRS 0–2) patients (mRS 3–6 group: 24% TICI 0, 1% TICI I, 1% TICI IIa, 37% TICI IIb, 1% TICI IIc, 34% TICI III; mRS 0–2 group: 29% TICI IIb, 4% TICI IIc, 67% TICI III; *p* < 0.0001). IVT intravenous thrombolysis, mRS modified Rankin scale, MT mechanical thrombectomy, TICI Thrombolysis in Cerebral Infarction

Reperfusion status (represented by mTICI) in the follow-up imaging was significantly different between dependent, bedridden, or dead patients (mRS score 3–6, *n* = 67) and patients with a good functional outcome (mRS score 0–2, *n* = 24, *p* < 0.0001; Fig. 3b). Higher rates of successful recanalization were detected in patients receiving at least one kind of recanalization therapy or the combination of both treatments compared to patients without any recanalization therapy (*p* < 0.0001, Fig. 3a). However, data (especially for individuals without any acute recanalization therapy) were incomplete. As a result, the group sizes varied greatly here (IVT only group *n* = 10, IVT + MT group *n* = 24, MT only group *n* = 30 vs. no IVT, no MT group *n* = 6).

### FEES Assessments During Ward Stay

Fifty-eight of the patients (60.42%) were treated by our speech and language therapists during their hospital stay, 16 (27.59%) of whom were examined by FEES. Thirty-eight of the patients (39.58%) were not examined because of persistent intubation/severe vigilance disorder. Of the 58 patients examined (+ /−FEES), 30 (51.72%) showed dysphagia and 28 (48.28%) did not. Of the 30 patients with neurogenic dysphagia, 11 patients (36.67%) were discharged or transferred with a nasogastric tube (and thus a poorer prognosis for full recovery of the dysphagia). Fifteen patients (50%) were at least partially to fully oralized. No information on the nutritional status of four patients (13.33%) was available at discharge. Among the 42 patients who did not undergo FEES but were alert enough for a clinical swallowing examination, 22 (52.4%) patients did not have adequate cooperation or alertness for FEES, 13 (31%) showed normal findings, and 7 (16.6%) exhibited abnormalities, though none required a nasogastric tube.

### Clinical Outcomes at Discharge

**Fig. 4:**
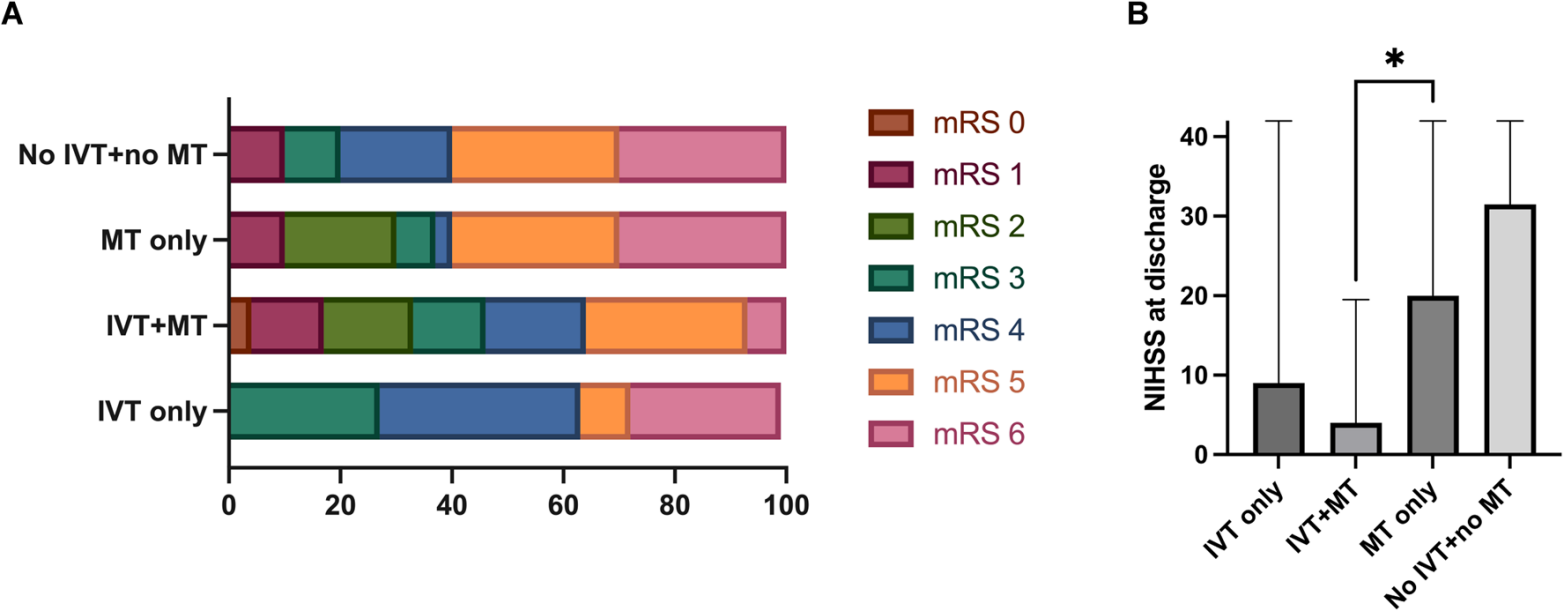
**a,** Frequency distribution of the neurological outcome based on mRS at discharge (IVT only group: *n* = 11; IVT + MT group: *n* = 45; MT only group: *n* = 30; no IVT + no MT group: *n* = 10; *p* < 0.001). **b,** median NIHSS score at discharge (IVT only group: 9 [IQR 2–35], *n* = 11; IVT + MT group: 4 [IQR 1–20], *n* = 45; MT only group: 20 [IQR 4–42], *n* = 30; no IVT + no MT group: 32 [IQR 2–42], *n* = 10; *p* = 0.0115). IQR interquartile range, IVT intravenous thrombolysis, mRS modified Rankin scale, MT mechanical thrombectomy, NIHSS National Institutes of Health Stroke Scale

Although not designed for therapy comparisons, our study found significant differences in functional outcomes. Patients treated with both IVT and MT showed better NIHSS scores at discharge (median 4, IQR 1–20, *n* = 45) compared to patients treated with IVT alone (median 9, IQR 2–35, *n* = 11), MT alone (median 20, IQR 4–42, *n* = 30), or no therapy (median 32, IQR 2–42, *n* = 10; *p* = 0.0115; Fig. 4b). The median mRS score at discharge was 4 for IVT (IQR 3–6, *n* = 11) or IVT + MT (IQR 2–5, *n* = 45) and 5 for MT alone (IQR 2–6, *n* = 30) or no therapy (IQR 4–6, *n* = 10; *p* = 0.0845; Fig. 4a). Among MT patients, those with prior IVT had significantly better mRS outcomes (Mann–Whitney *U*-test between IVT + MT [*n* = 45] and MT only [*n* = 30] groups, actual median rank difference 1, *p* = 0.046).

In the univariate analysis (Spearman correlation), the NIHSS and GCS scores at admission, prior mRS score, thrombus length, age, and success of recanalization assessed by the TICI score were significantly associated with functional dependence at discharge as measured by mRS and BI (Supplemental Table S3). In a next step, we added a multivariable median regression model that (consistent with the results of the univariate analysis) identified recanalization success, as measured by the TICI score, and the GCS score at admission as independently associated with functional outcome at discharge, as measured by mRS and BI, respectively (Table 3). When comparing patients with favorable outcome (mRS score ≤ 2) and patients with poor outcome (mRS score ≥ 3), we identified a detrimental impact of ataxia and thalamic infarcts (Table 4).

**Table 3:** Multivariable median regression model for functional outcomes

Predictors	Estimates	CI	*p* value
mRS at discharge (*n* = 89, *R*^2^ = 0.397)			
Age	0.00	− 0.04 to 0.04	0.852
Thrombus length	− 0.00	− 0.06 to 0.06	0.952
pcASPECTS	− 0.00	− 0.22 to 0.21	0.978
Prior mRS	0.03	− 0.41 to 0.48	0.880
NIHSS at admission	0.00	− 0.07 to 0.08	0.906
GCS at admission	− 0.16	− 0.32 to 0.00	0.053
TICI	− 0.37	− 0.61 to − 0.14	0.002
BI at discharge (*n* = 89, *R*^2^ = 0.334)			
Age	− 0.06	− 0.66 to 0.55	0.848
Thrombus length	− 0.03	− 1.16 to 1.11	0.964
pcASPECTS	− 0.02	− 2.11 to 2.07	0.984
Prior mRS	2.23	− 2.76 to 7.22	0.377
NIHSS at admission	− 0.35	− 1.00 to 0.30	0.290
GCS at admission	2.40	0.05 to 4.75	0.046
TICI	2.99	− 0.54 to 6.53	0.096

BI, Barthel Index; CI, confidence interval; GCS, Glasgow Coma Scale; mRS, modified Rankin scale; NIHSS, National Institutes of Health Stroke Scale; pcASPECTS, posterior circulation Alberta Stroke Program Early Computed Tomography Score; TICI, Thrombolysis in Cerebral Infarction

**Table 4:** Association of case characteristics with functional outcome at discharge

	Patients with mRS ≤ 2 (*n* = 25)	Patients with mRS ≥ 3 (*n* = 71)	Odds ratio (95% CI)	*p* value
*Sex*				
Female	10	30	1.096 (0.396–3.137)	1
Male	15	41		
*TOAST etiology*				
LAA	4	20		0.277
CE	13	40		
SVO	0	0		
Other determined	3	4		
Undetermined	5	7		
*Risk factors*				
Hypertension	20	59	0.748 (0.207–3.09)	0.7561
Diabetes mellitus	4	22	0.41 (0.091–1.427)	0.1918
Hypercholesterolemia	17	54	0.555 (0.178–1.799)	0.2782
Atrial fibrillation	14	37	1.168 (0.425–3.27)	0.8178
Smoking	6	13	0.924 (0.228–3.474)	1
*Symptoms*				
Impaired vigilance	14	55	2.669 (0.9–7.85)	0.0681
Neuropsychological changes	2	14	0.345 (0.035–1.696)	0.221
Aphasia	24	7	0.384 (0.008–3.253)	0.676
Dysarthria	20	57	0.705 (0.19–2.955)	0.5413
Dysphagia	15	44	0.98 (0.329–3.087)	1
Hemiparesis	11	41	0.502 (0.176–1.396)	0.1613
Hemisensory loss	4	12	0.858 (0.181–3.266)	1
Ataxia	16	22	3.344 (1.171–10.126)	0.01662
Vertigo	10	19	1.571 (0.53–4.556)	0.4511
Bulbar symptoms	3	7	1.148 (10.176–5.603)	1
Hemianopia	2	12	0.388 (0.039–1.96)	0.3338
Pupillomotor	8	36	0.461 (0.152–1.3)	0.1608
Oculomotor	15	33	1.628 (0.589–4.668)	0.3543
Babinski sign	9	24	1.077 (0.362–3.06)	1
*Vessel occlusions*				
Right P1-segment	19	41	2.298 (0.763–7.9)	0.1495
Left P1-segment	17	57	0.526 (0.169–1.7)	0.2688
Right SUCA	11	34	0.856 (0.3–2.351)	0.8178
Left SUCA	10	42	0.464 (0.162–1.282)	0.1091
*Infarct locations*				
Thalamus infarct	1	19	0.11 (0.0026–0.8009)	0.0198
Occipital infarct	2	13	0.378 (0.038–1.88)	0.3393
Mesencephalic infarct	5	17	0.767 (0.195–2.562)	0.7855
Pontine infarct	3	14	0.538 (0.09–2.1)	0.5454
Cerebellar infarct	7	23	0.779 (0.239–2.318)	0.8029

CE, cardioembolic; CI, confidence interval; LAA, large artery atherosclerosis; mRS, modified Rankin scale; SUCA, superior cerebellar artery; SVO, small vessel occlusion; TOAST, Trial of Org 10,172 in Acute Stroke Treatment

## Discussion

We aimed to identify clinical and imaging factors influencing clinical decisions and outcomes in TOBS. On admission, the majority of our patients showed a reduction in vigilance with some additional bulbar symptoms, hemiparesis, and deficits in pupillary and oculomotor function, which in combination emerged as a guiding finding in the diagnosis of TOBS. Many also exhibited a Babinski sign, suggesting pyramidal tract involvement and more severe stroke syndromes. We were able to show that the initial severity of the syndrome (reduced GCS score and higher NIHSS score) and the presence of ataxia was associated with a poorer outcome at discharge or transfer.

In line with the known pitfalls of posterior circulation strokes [7, 9], almost half of the patients showed no infarcts in the first imaging. Infarction of the thalamus was associated with poor outcome. The presumed mesencephalic involvement at initial imaging (usually indicative of more extensive occlusion) was not associated with poor outcome. We confirmed that MRI was superior to CT for the detection of infarcts in the posterior circulation [6, 21]. In one case, Castaigne syndrome [12] caused TOBS, a rare occurrence. More commonly, rupture or direct compression of the brainstem structures leads to symptoms in unruptured basilar apex aneurysms [22–24]. The treatment of these aneurysms may also lead to thromboembolic complications that are associated with TOBS, especially in the presence of an artery of Percheron [25–27].

Most patients received both IVT and MT, and successful recanalization measured by the TICI score was significantly associated with better functional outcome, consistent with recent reports linking reperfusion therapy to lower 1-year mortality after TOBS [14]. Koh et al. also highlighted that lack of reperfusion in patients treated primarily with MT led to early neurologic deterioration [28]. Typical for posterior circulation strokes, the time windows from symptom onset/last seen well to arrival at our comprehensive stroke center were relatively long compared to the average time window for the anterior circulation, influenced by delays both in transfer and direct presentation [30, 31]. Best functional outcomes (measured by mRS and BI at discharge) were found in patients receiving both IVT and MT.

The study primarily focused on mRS and BI outcomes at discharge, emphasizing mobility and daily independence, but dysphagia and the associated cost restrictions also emerged as critical due to its impact on quality of life, risk of choking, susceptibility to pulmonary infections, and costs [32, 33]. Initially, 61% of the patients exhibited dysphagia, with 50% improving at discharge or transfer. However, 11 patients required nasogastric tubes, indicating poor recovery prospects. Early detection and intervention are vital to prevent complications such as aspiration, pneumonia, and malnutrition, along with extended hospital stays and poor discharge outcomes. Dysphagia also affects psychological well-being, often linked to depression and reduced independence [34–37]. Besides cranial nerve impairment and affection of swallowing central pattern generators with extended brain stem lesions, the underlying cause of dysphagia was mostly explained by reduced vigilance, which was restored in many of the observed cases over time. Because our data are limited to discharge or transfer, further studies on dysphagia after TOBS are needed given its significant impact on stroke survivors’ quality of life.

The present work adds descriptive data from a highly selective and underrepresented patient cohort, but there are notable limitations. Firstly, the study’s retrospective design and small subgroup sizes, especially for patients receiving no therapy or IVT alone, limit generalizability. Second, data gaps, particularly in time windows for secondary transfers, hinder comparisons between direct and transferred patients, treatment modalities, and the impact of transfer delays. Furthermore, 40% of the patients were admitted analgosedated or intubated for MT evaluation, introducing a selection bias, as these patients were specifically assigned for higher-level care at our comprehensive stroke center. Confounding factors such as previous condition (age, previous illnesses, preexisting disability) and the extent of initial infarcts were not controlled for, which affects the validity of comparisons. Moreover, MT was sometimes used as a rescue therapy in less favorable conditions. Using a radiology database presents further limitations due to potential inconsistencies in imaging quality and data from different machines and protocols. However, the combination of clinical and radiology databases provided sufficient information for linking clinical data. Patients identified using the term “distal basilar artery syndrome” who had spontaneously or successfully recanalized distal basilar artery occlusions through IVT were included. The absence of FEES for some patients due to high vigilance disorders among patients also contributes to a bias in generalizability. Lastly, the study is limited to discharge data, preventing long-term outcome analysis beyond short-term prognosis. Further validation through prospective studies over a longer period, especially for mildly affected patients with TOBS, is needed. Despite these limitations, our study offers valuable insights into the clinical characteristics and short-term functional outcomes of TOBS.

## Conclusions

In patients with TOBS, impaired consciousness resulting in consecutively increased initial stroke severity as measured by the NIHSS, implicated treatment decisions, and vascular recanalization success as assessed by the TICI score were highly associated with functional outcome at discharge.

## Source of Support

No specific grant or financial support was received for this study.

## Supplementary Information

Below is the link to the electronic supplementary material.

Supplementary file1 (DOCX 22 KB)

## Data Availability

All data will be available upon reasonable request.
