# Supplementary material for: Symptoms, Imaging Features, Treatment Decisions, and Outcomes of Patients with Top of the Basilar Artery Syndrome: Experiences from a Comprehensive Stroke Center
Source: Neurocrit Care. 2025 Feb 7;43(1):69–79. doi: 10.1007/s12028-025-02219-y (PMC12321678; doi:10.1007/s12028-025-02219-y)
Supplement: Supplementary file 1 — Supplementary file1 (DOCX 22 KB) [file 12028_2025_2219_MOESM1_ESM.docx]

**Supplemental Material**

**Results**

**Treatment decisions - secondary prevention**

Secondary prevention with oral anticoagulation (OAC) in patients without prior antiplatelet or anticoagulant therapy was initiated in all surviving patients depending on whether atrial fibrillation (AF) was registered during ward stay, which applied to 12 patients (31%). 17 of the patients (44%) without prior antiplatelet or anticoagulant therapy were put on antiplatelet monotherapy, while in 4 patients (10%) dual antiplatelet therapy (DAPT) was initiated, either because they received a vertebral or basilar stent (3 patients) or because persistent intracranial stenoses were detected (one patient). Of the patients with prior antiplatelet therapy, 10 patients (30%) maintained the secondary prevention unchanged, while 11 patients (33%) were switched to OAC following the detection of AF. In 8 patients (24%), antiplatelet therapy was escalated to DAPT, 2 for acute coronary events requiring stenting during ward stay, one for a vertebral stent. Of the patients with prior OAC, 4 patients (21%) maintained the secondary prevention unchanged, while 8 patients 42% were switched to a different OAC. One patient with prior OAC, the OAC was discontinued in favor of starting DAPT following a basilar stent, but OAC was recommended for the future.

**Table S1** Thrombus characteristics.

| **thrombus hallmarks** | |  |  |  |  |  |  |
| --- | --- | --- | --- | --- | --- | --- | --- |
|  |  | **n** | **%** | **mean** | **SD** | **median** | **IQR** |
|  | total | 96 |  |  |  |  |  |
| thrombus length (mm) | |  |  | 9.59 | 6.5 | 8 | 5-12 |
| involvement further arteries | left P1 | 74 | 77% |  |  |  |  |
|  | right P1 | 60 | 63% |  |  |  |  |
|  | left SUCA | 52 | 54% |  |  |  |  |
|  | right SUCA | 45 | 47% |  |  |  |  |

Abbreviations: P1 = P1-segment of the posterior cerebral artery, SUCA = superior cerebellar artery.

**Table S2** Time windows for the different treatment groups including the time for transfer between centers.

|  | **IVT only** | **IVT+MT** | **MT** | **no IVT**  **/no MT** |
| --- | --- | --- | --- | --- |
| overall time from symptom onset to arrival at **our** comprehensive stroke center (time in minutes, data available in n (%) of the cases) | 285.6**±**56.57 n=7 (63.63%) | 197.8**±**16.98 n=38 (84.44%) | 286.3**±**58.39 n=19 (63.33%) | 329.8**±**104.1 n=6 (60%) |
| presentation directly to **our** comprehensive stroke center (time in minutes, data available in n of the cases) | 66  n=1/3 | 100.9±19.11 n=13/16 | 203±69.57 n=12/18 | 84.5±23.5  n=2/4 |
| transfers from primary stroke center (time in minutes, data available in n of the cases) | 322.2±51.04,  n=6/8  *IVT initiated before transport | 248.2±16.48  n=25/29  *IVT initiated before transport | 427.6±85.35  n=7/9 | 452.5±109.4  n=4/6 |

Note: In the MT-group, 3 of the cases were in-hospital strokes, where time windows were not applicable.

**Table S3** Treatment decisions.

| **reasons against IVT** | **MT only/ no IVT, no MT group together**  (number of patients) |
| --- | --- |
| pre-existing (oral) anticoagulation | n=13 (32.5%) |
| unknown or already exceeded time window | n=12 (30%) |
| increased extracerebral bleeding risk e.g. through malignancies, larger surgeries/interventions or previous life-threatening gastrointestinal bleedings shortly before the stroke | n=6 (15%) |
| arteriosclerotic/dissecting process in the vertebral artery that had already led to infarctions 1-2 days previously | n=3 (7.5%), one with a previous on-label IVT |
| initial (partially extended) infarct demarcation in the very first imaging | n=21 (52.5%)  MT-only group: n=15 (50%)  no IVT+no MT group: n=6 (60%) |
| reason unknown | n=3 (7.5%) |
| **reasons against MT** | **no IVT, no MT group** |
| expected technical/interventional nature (no thrombectomy target or too high periinterventional risk) | n=3 (7.5%) |

**Table S4** Nonparametric spearman correlations for functional outcome.

|  | **n** | **rho** | **p value** | **Benjamini-Hochberg adjusted p value** |
| --- | --- | --- | --- | --- |
| **mRS discharge** | | | | |
| GCS at admission | 96 | -0.4547 | <0.0001 | **0.0002** |
| NIHSS at admission | 96 | 0.5206 | <0.0001 | **0.0002** |
| TICI | 91 | -0.4959 | <0.0001 | **0.0002** |
| prior mRS | 96 | 0.3306 | 0.001 | **0.002** |
| thrombus length | 95 | 0.2655 | 0.0093 | **0.01** |
| age | 96 | 0.23 | 0.0228 | **0.03** |
| pcASPECTS | 95 | -0.1362 | 0.188 | 0.188 |
| **BI discharge** | | | | |
| GCS at admission | 96 | 0.5283 | <0.0001 | **0.0002** |
| NIHSS at admission | 96 | -0.5567 | <0.0001 | **0.0002** |
| TICI | 91 | 0.4311 | <0.0001 | **0.0002** |
| prior mRS | 96 | -0.2230 | 0.029 | **0.0343** |
| thrombus length | 95 | -0.2723 | 0.0076 | **0.0133** |
| age | 96 | -0.2224 | 0.0294 | **0.0343** |
| pcASPECTS | 95 | 0.1803 | 0.0805 | 0.0805 |
